# Partial autoamputation of an ovary following adnexal torsion with preserved ovarian function in a reproductive-age woman: a case report and literature review

**DOI:** 10.1186/s12905-026-04597-7

**Published:** 2026-06-11

**Authors:** Kino Hayashi, Hideaki Tsuyoshi, Akari Nakamori, Michio Watanabe, Yasuo Hayashi, Yuka Hattori, Makoto Orisaka, Yoshio Yoshida

**Affiliations:** 1 Department of Obstetrics and Gynecology, Sugita Genpaku Memorial Obama Municipal Hospital, 2-2 Otecho, Obama-shi, Fukui, 917-0078 Japan; 2 Department of Obstetrics and Gynecology, Faculty of Medical Sciences, University of Fukui, 23-3 Shimoaizuki, Matsuoka, Eiheiji-cho, Yoshida-gun, Fukui, 910-1193 Japan; 3 Department of Surgery, Sugita Genpaku Memorial Obama Municipal Hospital, 2-2 Otecho, Obama-shi, Fukui, 917-0078 Japan

**Keywords:** Ovarian tumor pedicle torsion, Autoamputated ovary, Laparoscopic surgery, Preserved ovary, Follicular development

## Abstract

**Background:**

Ovarian autoamputation is a rare consequence of adnexal torsion. Preoperative diagnosis is challenging, and ovarian and tubal function on the affected side is rarely preserved once autoamputation has occurred.

**Case:**

A 29-year-old woman with a known benign-appearing left ovarian tumor presented with acute lower abdominal pain. Imaging studies suggested torsion of the ovarian tumor pedicle, and emergency laparoscopic surgery was performed. Intraoperatively, a necrotic, fist-sized mass consistent with an autoamputated ovarian tumor was identified in the pouch of Douglas, while the remaining ovary and fallopian tube were preserved. Histopathological examination revealed hemorrhagic infarction and necrosis of the tumor, with residual ovarian stroma containing follicles. Postoperative follow-up demonstrated normal follicular development and resumption of ovulatory function, confirming preserved ovarian function on the affected side.

**Conclusion:**

Unlike previous reports of ovarian autoamputation, this rare case of partial ovarian autoamputation suggests that spontaneous detachment of necrotic tissue does not inevitably result in the complete loss of ipsilateral ovarian function. However, in reproductive-age women, early surgical intervention should be strongly considered when adnexal torsion is suspected, even in the absence of definitive imaging findings, to maximize the potential for preserving ovarian function.

## Background

Ovarian pedicle torsion is a gynecological emergency, accounting for 2.7% of acute gynecologic conditions and ranking fifth among such emergencies [1, 2]. Although it occurs across all age groups, it predominantly affects women of reproductive age, with 30% of cases occurring in individuals younger than 20 years [3, 4]. The condition arises when the adnexa twists around the axis of the ovarian and infundibulopelvic ligaments, leading to compromised vascular flow [3]. While torsion may occur in normal ovaries, the risk increases substantially with adnexal masses exceeding 5 cm in diameter [5].

An autoamputated ovary is a rare clinical entity, defined as the spontaneous detachment of a normal ovary or ovarian tumor following prolonged pedicle torsion [6]. Its incidence is approximately 1 in 11,421 cases [7]. Sustained torsion results in ischemia and necrosis, leading to the separation of necrotic adnexal tissue due to complete disruption of the distal blood supply [8, 9]. The diagnosis is typically made intraoperatively upon finding an absent ovary with a blindly ending fallopian tube lacking fimbriae or an identifiable infundibulum [10, 11]. Preoperative diagnosis remains challenging in the absence of definitive imaging criteria. After autoamputation, therapeutic options are limited, and irreversible loss of ovarian function is generally assumed, raising concerns regarding fertility [6, 10].

We report a case in which ovarian pedicle torsion resulted in the spontaneous detachment of an ovarian tumor with partial autoamputation. Residual ovarian tissue with preserved function remained on the affected side, representing an exceptionally rare presentation of partial ovarian autoamputation in a reproductive-age woman with maintained ipsilateral ovarian function. This case highlights the importance of early surgical intervention for suspected adnexal torsion in this population.

## Case presentation

A 29-year-old woman, gravida 1 para 0, abortion 1, presented with a regular menstrual cycle occurring every 30 days and lasting 4–7 days. The patient’s medical history included pelvic peritonitis due to *Chlamydia trachomatis* and conservatively treated acute appendicitis at the age of 27, but she had no other inflammatory diseases, such as endometriosis. She had no history of medication use or nutraceutical supplementation.

**Fig. 1:**
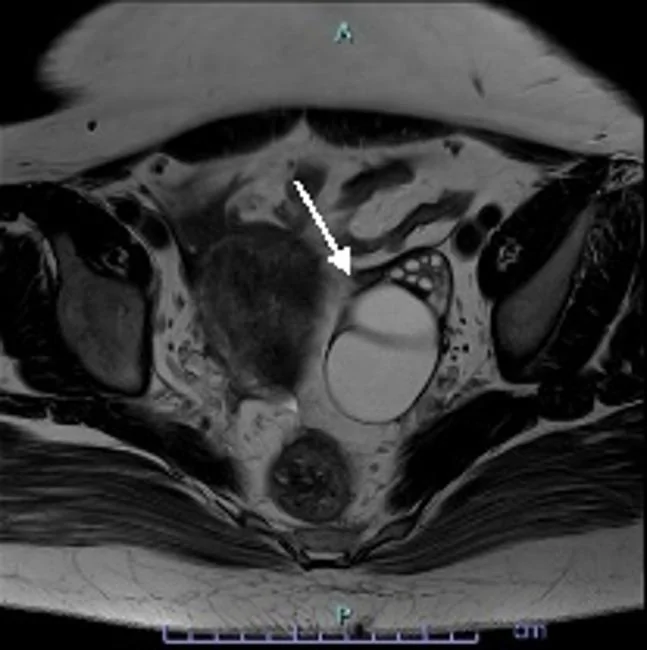
Axial T2-weighted MRI reveals a 6.5 cm multilocular tumor in the left ovary (arrow). No solid components or mural thickening are present

A multilocular left ovarian tumor measuring 6.5 cm in diameter was detected during a routine gynecological examination by her previous physician. The patient requested surgical management and was referred to our department. Contrast-enhanced magnetic resonance imaging (MRI) demonstrated a 6.5 cm multilocular cystic mass in the left ovary, showing low signal intensity on T1-weighted images and high signal intensity on T2-weighted images. No solid components or mural thickening suggestive of malignancy were identified, and the lesion was suspected to be a mucinous cystadenoma (Fig. 1).

The patient had severe obesity (body mass index, 37 kg/m^2^; height, 156 cm; weight, 90 kg) and poorly controlled type 2 diabetes mellitus (hemoglobin A1c (HbA1c), 9.2%). Considering the increased perioperative risk, surgical intervention was deferred, and medical optimization was prioritized.

The tumor was monitored at three-month intervals, during which no change in size was observed. Nine months after the initial consultation, the patient presented to the emergency department with persistent left lower abdominal pain that had begun the previous evening. She reported experiencing intermittent episodes of similar pain in the past, which had resolved spontaneously. Her last menstrual period had occurred 20 days prior and lasted four days, and her most recent sexual intercourse had been one week earlier.

On presentation, torsion of the known left ovarian tumor was suspected, and the patient was referred to our department. Her vital signs were stable: heart rate, 119 beats/min; blood pressure, 110/80 mmHg; respiratory rate, 16/min; body temperature, 36.9 °C; and oxygen saturation, 98% on room air. On pelvic examination, marked tenderness was noted in the left lower abdomen, and a soft mass was palpated in the left adnexal region. Mild uterine tenderness and persistent serosanguinous vaginal discharge were observed on speculum examination.

Transvaginal ultrasonography demonstrated a multilocular cyst measuring 7.5 × 5.5 cm in the left adnexal region. No abnormalities were observed in the endometrium or right adnexa. Laboratory tests showed mild inflammatory changes, with a white blood cell count of 11,260/µL and C-reactive protein level of 3.496 mg/dL. Tumor markers carcinoembryonic antigen (CEA) and carbohydrate antigen 19 − 9 (CA19-9) were within normal limits (CEA < 1.00 ng/mL; CA19-9, 3.4 U/mL), whereas cancer antigen 125 (CA125) was mildly elevated (54.3 U/mL). The pregnancy test result was negative.

**Fig. 2:**
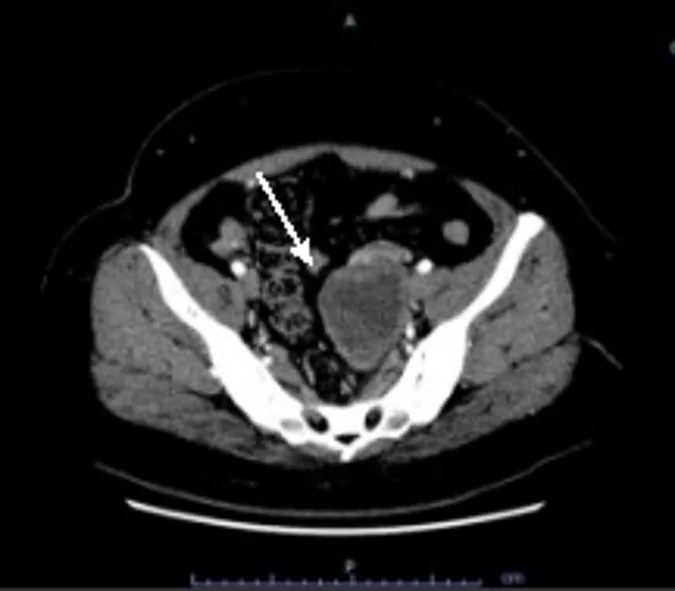
Contrast-enhanced CT shows a 7.5 cm multilocular cystic lesion in the left ovary, characterized by poor contrast enhancement, and suggesting blockage of blood flow leading to ischemia and necrosis of ovarian tissue (arrow)

**Fig. 3:**
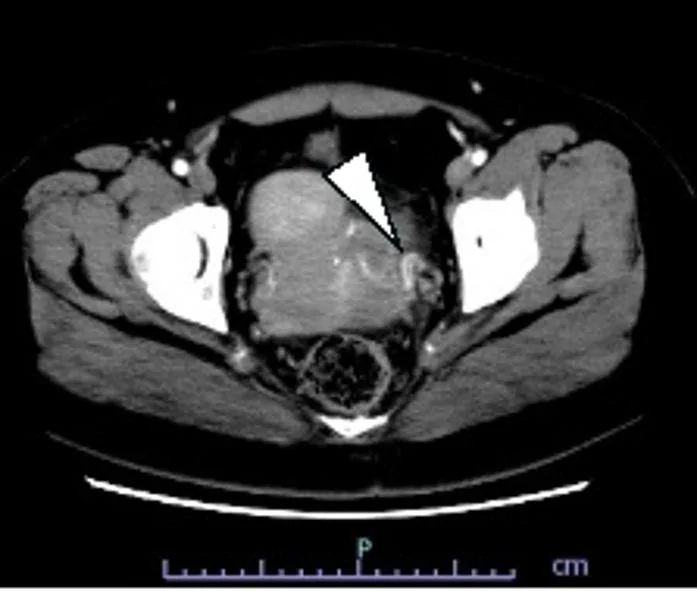
Contrast-enhanced CT demonstrates the ovarian artery and vein in a spiral shape, termed the whirlpool sign, suggesting that the ovarian vessels are twisted (arrowhead)

Contrast-enhanced computed tomography (CT) demonstrated a 7.5 cm multilocular cystic mass in the left ovary, with mural thickening and poor contrast enhancement (Fig. 2). No mural nodules or solid components were identified, and there were no findings suggestive of malignancy. A whirlpool sign involving the left ovarian artery and vein was observed, consistent with adnexal torsion (Fig. 3). No abnormalities were detected in other intra-abdominal organs.

**Fig. 4:**
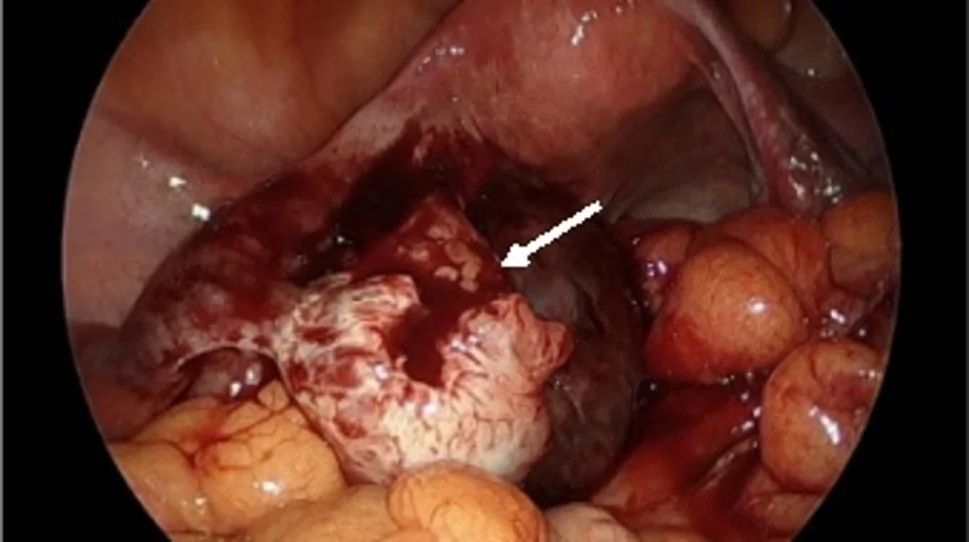
Examination of the abdominal cavity revealed a laceration and slight enlargement of the left ovary, with adherent blood clots and mild oozing (arrow). No active bleeding or necrotic changes were observed

**Fig. 5:**
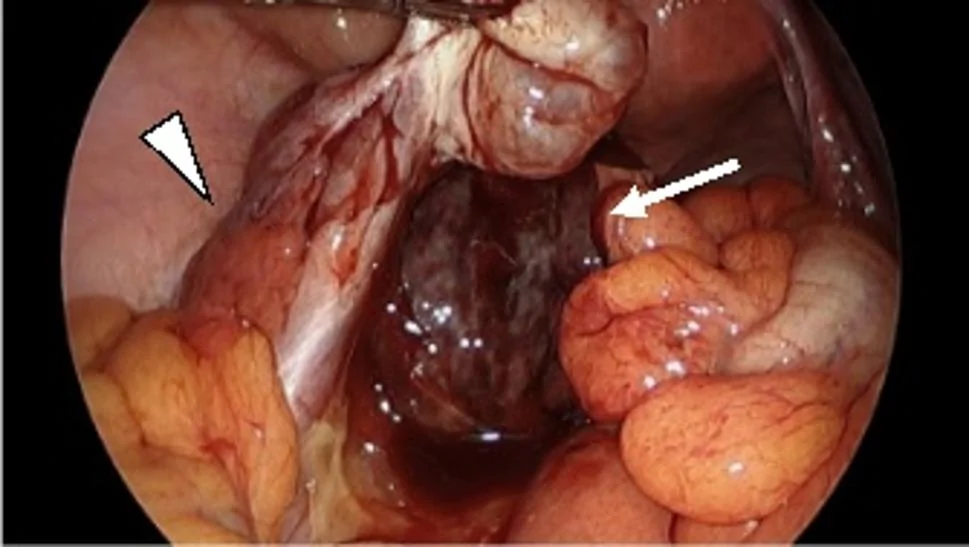
Assessment of the abdominal cavity revealed congestion and edema of the left fallopian tube, proper ovarian ligament, and infundibulopelvic ligament (arrowhead). A black mass approximately fist-sized was identified in the pouch of Douglas (arrow)

Based on these findings, emergency laparoscopic surgery was performed under general anesthesia. Intraoperatively, the left ovary was mildly enlarged (approximately 4 cm) compared with the right ovary. A laceration of approximately 3 cm was observed on the surface, with adherent blood clots and slight oozing. No active bleeding was noted. There were no findings suggestive of necrosis based on color or gross appearance (Fig. 4). The left fallopian tube, proper ovarian ligament, and infundibulopelvic ligament were markedly congested and edematous. A black, fist-sized mass was identified in the pouch of Douglas (Fig. 5).

**Fig. 6:**
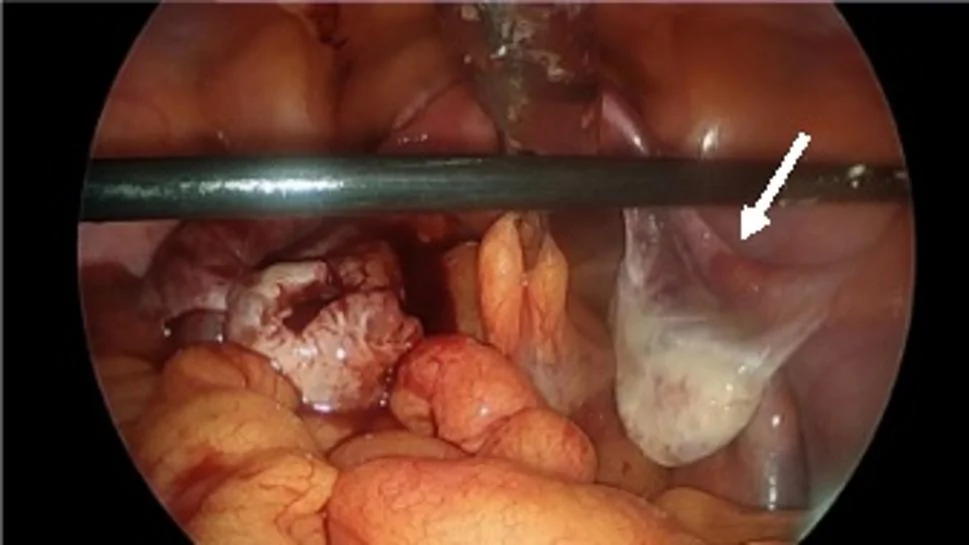
Intra-abdominal findings. The uterus, contralateral right ovary, and fallopian tube (arrow) appear normal

**Fig. 7:**
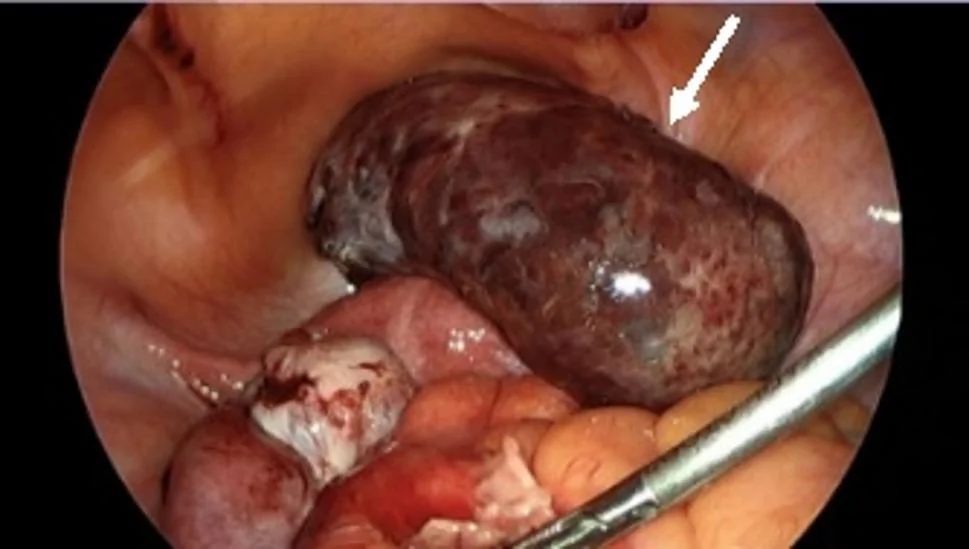
Intra-abdominal findings. The mass in the pouch of Douglas shows no adhesions to the surrounding tissues or communication with adjacent organs (arrow)

The uterus, right ovary, and right fallopian tube appeared normal (Fig. 6), and no ascites or additional intra-abdominal abnormalities were noted. The mass in the pouch of Douglas exhibited no adhesions to the surrounding tissues and did not communicate with adjacent organs (Fig. 7). After confirming hemostasis in the residual left ovary, the mass was excised, and the procedure was completed laparoscopically. The total operative time was 1 h 33 min, with an estimated blood loss of 100 mL.

**Fig. 8:**
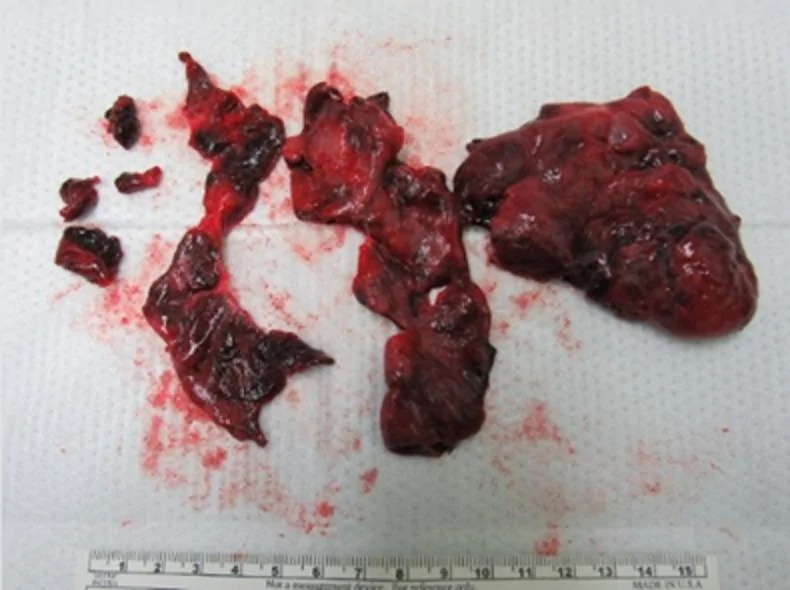
Gross examination of the excised mass in the pouch of Douglas shows a smooth cyst wall devoid of nodules or solid components

Gross examination of the excised specimen revealed a cyst containing brown to reddish-brown serosanguineous fluid. The cyst wall was smooth, without nodules or solid components, and the boundary between the cyst wall and ovarian stroma was poorly defined (Fig. 8).

**Fig. 9:**
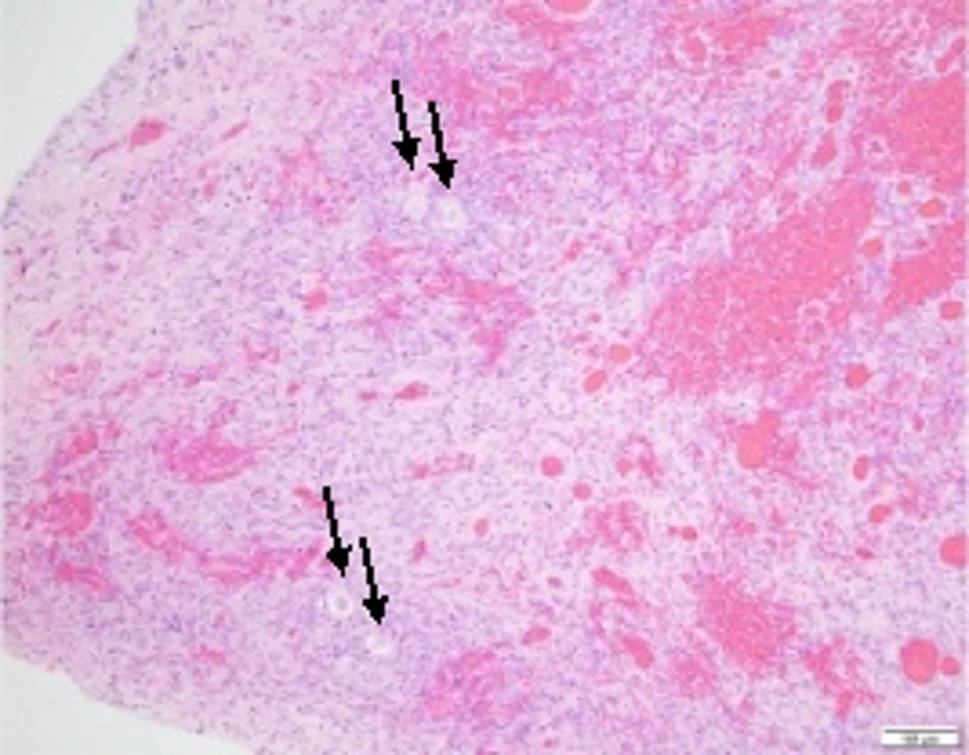
Hematoxylin-eosin staining of the pathological specimen reveals hemorrhagic infarction and necrosis due to edema throughout the mass. Ovarian stroma and follicles were also identified in focal regions (arrows) (magnification, × 100)

Histopathological examination showed widespread hemorrhagic infarction and edema, consistent with ischemic necrosis. Most of the cyst wall was necrotic, preventing evaluation of the epithelial lining. Focal neutrophilic infiltration suggested an acute inflammatory process, and marked fibrin deposition on the cyst surface indicated acute disruption of blood flow. Notably, ovarian stromal tissue containing follicles was identified in a localized area of the cyst wall (Fig. 9). Based on the pathological findings and clinical course, the mass in the pouch of Douglas was diagnosed as a necrotic ovarian tumor resulting from partial autoamputation secondary to torsion of the left ovarian tumor pedicle.

**Fig. 10:**
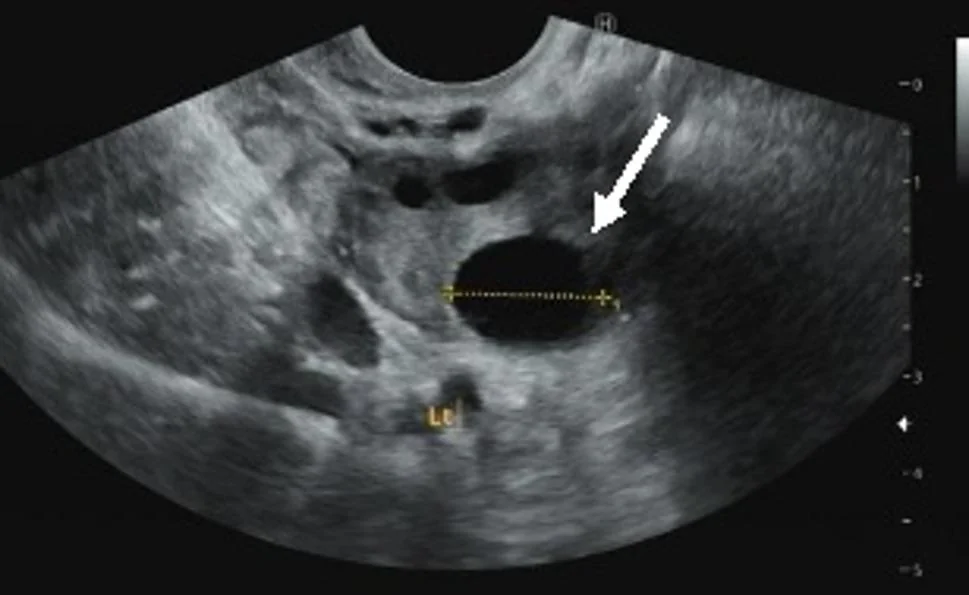
Transvaginal ultrasound performed one month after surgery shows follicular development in the left ovary, the affected side (arrow)

The postoperative course was uneventful, with no signs of infection. The patient was discharged on postoperative day 6. One month after surgery, transvaginal ultrasonography showed follicular development in the affected ovary (Fig. 10). At the 6-month follow-up visit, ovulatory function in the affected ovary and normal menstruation were observed, with no evidence of tumor recurrence.

## Discussion

Ovarian autoamputation is a rare but significant sequela of prolonged adnexal torsion. It represents the terminal stage of ischemic injury, resulting in irreversible loss of ovarian and tubal function on the affected side. This case challenges the conventional understanding by demonstrating that partial autoamputation can occur with preservation of residual ovarian tissue and function, thereby expanding understanding of adnexal torsion pathophysiology and reproductive implications.

The preoperative diagnosis of adnexal torsion and autoamputation remains challenging because of nonspecific symptoms and limited imaging accuracy. Transvaginal ultrasonography is the primary diagnostic modality and is often combined with laboratory testing and tumor markers to exclude malignancy [8, 12]. However, preserved ovarian blood flow on Doppler imaging does not exclude torsion, as normal flow is observed in up to 60% of cases [3, 6]. Inflammatory markers, such as leukocyte count and C-reactive protein, may be elevated but lack specificity [9, 13]. Contrast-enhanced CT is used in emergencies, but its sensitivity to detecting torsion is low [14]. On the other hand, MRI has high resolution for soft tissue, and is particularly specific to torsion in adnexal tumors [15]. Nevertheless, MRI is often impractical in emergency settings because of limited availability, cost, and time constraints [16]. In our case, contrast-enhanced CT demonstrated decreased enhancement of the left ovarian tumor and a whirlpool sign of the left ovarian vessels, suggesting torsion; however, surgery revealed no adnexal torsion, with the viable left ovary remaining in its normal position and the necrotic mass isolated in the pouch of Douglas. Although ultrasonography and CT identified a tumor adjacent to the normal ovary, they failed to clarify the precise anatomical relationship and blood flow, making preoperative diagnosis of autoamputation impossible. These discrepancies between imaging and intraoperative findings likely reflect the dynamic nature of torsion and autoamputation, highlighting the continued importance of clinical judgment because no imaging modality can reliably establish the diagnosis preoperatively [3].

The differential diagnosis of a mass isolated in the Douglas pouch includes intra-abdominal masses (such as peritoneal carcinoma) and ovarian tumors that have undergone auto-excision due to torsion or ovulation. Distinguishing the former is important, and preoperative imaging does not show signs of torsion. Intraoperatively, the tumor may infiltrate surrounding tissues and show dissemination to the ovary, but there is no ovarian rupture. These findings can be useful for differential diagnosis. In contrast, in the latter category, both preoperative imaging and intraoperative findings show signs of torsion or rupture in the ovary, and, as in this case, complete distinction is not possible. Pathological findings can help differentiate between the tumor and normal ovarian tissue, but in cases such as this one, where the epithelial component is completely necrotic, definitive diagnosis may still be difficult.

To contextualize this case, we conducted a comprehensive review of published case reports of autoamputated ovaries in women of reproductive age (Table 1). A literature search was conducted on PubMed for the period from 2016 to 2025 using the search terms ‘auto-amputation of the ovary’ and ‘wandering ovary’. All retrieved articles were relevant to ovarian amputation, and no papers were excluded from this review except for two cases in postmenopausal women. Fourteen cases were identified [1, 8, 9, 13, 17–25].

**Table 1:** Literature review of autoamputated ovaries in women of reproductive age, published between 2016 and 2025

No.	Year	Study	Age (years)	Symptoms	Tumor diameter (cm)	Pathology	Side	Ovarian status	Tubal status	Ectopic location	Function of the affected adnexa
1	2016	Murphy [17]	34	Infertility	–	Hydrosalpinx	R	Intact	Partial amputation	Free	Lost
2	2017	Kim [18]	34	Mass	6	MCT	L	Complete amputation	Complete amputation	Free	Lost
3	2017	John [19]	32	Sterilization	6	MCT	L	Complete amputation	Partial amputation	Pouch of Douglas, rectum	Lost
4	2017	Atici [9]	16	Abd pain	2	MCT	R	Complete amputation	Partial amputation	Free	Lost
5	2019	Durous [20]	26	Low back pain	3	Fibroma	R	Complete amputation	Complete amputation	Free	Lost
6	2021	Daccache [21]	42	Mass	2	MCT	L	Complete amputation	Unknown	Free	Lost
7	2022	Habek [13]	24	Abd pain	7	Simple cyst	L	Complete amputation	Complete amputation	Free	Lost
8	2022	Atileh [1]	26	Abd pain	13	Endometrioma	L	Partial amputation	Complete amputation	Free	Lost
9	2023	Gorginzadeh [22]	14	Mass	10	MCT	L	Complete amputation	Complete amputation	Within the omentum	Lost
10	2023	Chaichian [23]	46	Amenorrhea	5	MCT	R	Complete amputation	Complete amputation	Free	Lost
11	2023	Ellison [24]	31	Abd pain	5	Unknown	L	Complete amputation	Complete amputation	Free	Lost
12	2024	Banoub [8]	28	Mass	6	Simple cyst	R	Intact	Partial amputation	Free	Lost
13	2025	Seyhan [25]	33	Abd pain	8	MCT	L	Complete amputation	Complete amputation	Pouch of Douglas	Lost
14	2025	Our case	29	Abd pain	7.5	Ovarian tissue with hemorrhagic infarction and necrosis	L	Partial amputation	Intact	Free	Preserved

*Abd* Abdominal, *L* Left, *MCT* Mature cystic teratoma, *R* Right

Similar to our case, abdominal or pelvic pain was the most common symptom, occurring in 50% of cases. Most diagnoses involved ovarian tumors, including mature cystic teratomas, whereas ovarian tissue with extensive hemorrhagic infarction and necrosis was not identified in these reports, in contrast to our case. Occasionally, the necrotic ovary detaches and may re-implant on structures such as the omentum or bowel, developing a collateral blood supply [6]. Post-autoamputation implantation occurred in 21% of cases, but our patient showed no adhesions or communication with other organs. In all cases, an autoamputated ovary was diagnosed only intraoperatively.

**Fig. 11:**
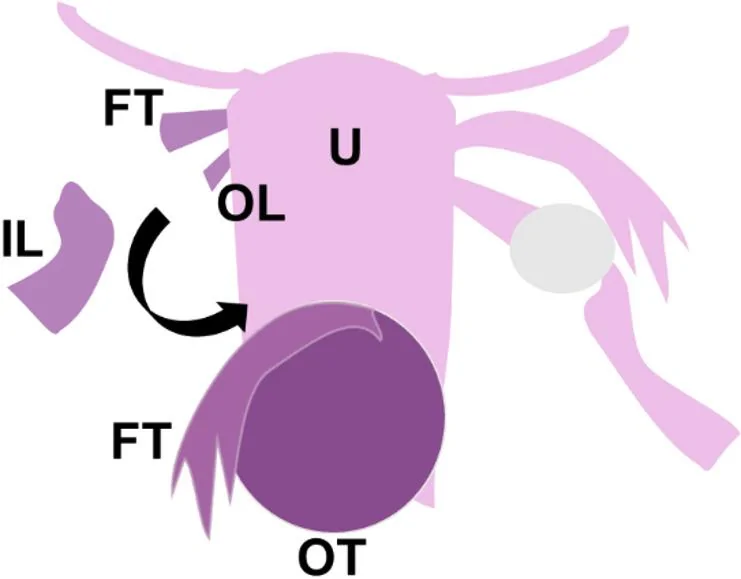
Schematic of the mechanism of autoamputated ovary described in previous reports. Twisting of the adnexa around the axis of the ovarian and infundibulopelvic ligaments leads to compromised vascular flow and detachment of the affected whole ovary and fallopian tube, compromising their function. U, uterus; OT, ovarian tumor; FT, fallopian tube; OL, ovarian ligament; IL, infundibulopelvic ligament

**Fig. 12:**
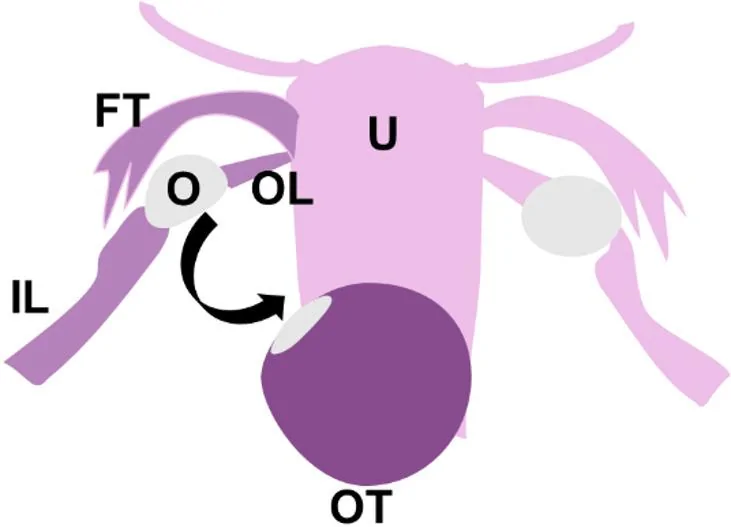
Speculative schematic of the mechanism of partial ovarian autoamputation in the present case. The adnexa twists around the axis between the margin of the normal ovary and the tumor, leading to compromised vascular flow and detachment of the tumor, while the remaining normal ovarian tissue is preserved, maintaining its function. U, uterus; O, ovary; OT, ovarian tumor; FT, fallopian tube; OL, ovarian ligament; IL, infundibulopelvic ligament

In previously reported cases, reproductive function on the affected side was irreversibly lost, with the fallopian tube being blind-ended or absent and the ovary absent in 71% of cases. Torsion of the ovarian and infundibulopelvic ligaments has been suggested to result in complete detachment of the adnexa, leading to loss of function (Fig. 11). These findings suggest that autoamputation is generally a devastating event for fertility. In the present case, continuity between the ovarian tissue fallopian tube was preserved, and postoperative follicular development confirmed maintenance of ovarian function. Torsion may have occurred between the normal ovary and the tumor, resulting in necrosis and detachment of the tumor while preserving residual ovarian function (Fig. 12). However, when an ovarian tumor arises at the distal tip of the ovary, even torsion of the adnexa around the axis of the ovarian ligament and infundibulopelvic ligament might compromise the blood supply to the tip, resulting in autoamputation of only the distal portion. The minimal bleeding observed was likely due to dry amputation after ischemia of the twisted tumor.

While this case report has the potential to significantly contribute to the field, certain limitations need to be considered. First, the literature review was confined to the period from 2016 to 2025. Because no cases resembling partial autoamputation of an ovary were identified in previously published case reports, and because this report aims to deepen understanding of this distinct pathological condition and its potential implications for reproduction, we limited the review period to the most recent ten years to avoid an excessive number of similar cases. Second, our proposed schematic of the autoamputation mechanism in the present case remains speculative. Therefore, we also proposed differential diagnoses other than ovarian tumors, as well as variations in the axis of torsion. Third, we currently have only six months of postoperative follow-up data. We will continue to monitor the patient for recurrence and assess ovarian function, including fertility outcomes.

## Conclusion

An autoamputated ovary is generally associated with irreversible loss of ipsilateral ovarian function; however, this case represents partial ovarian autoamputation with maintained ipsilateral ovarian function. Although autoamputation is rare, clinicians should be aware of the potential for atypical clinical courses and provide careful counseling to patients, as well as prompt surgical intervention when torsion of an ovarian tumor pedicle is suspected in reproductive-age women.

## Data Availability

The data supporting the findings of this study are available within the article.

## Abbreviations

HbA1c: Hemoglobin A1c
MRI: Magnetic resonance imaging
CT: Computed tomography
CEA: Carcinoembryonic antigen
CA19-9: Carbohydrate antigen 19 − 9
CA125: Cancer antigen 125
